# A reappraised meta-analysis of the genetic association between vitamin D receptor *Bsm*I (rs1544410) polymorphism and pulmonary tuberculosis risk

**DOI:** 10.1042/BSR20170247

**Published:** 2017-06-07

**Authors:** Mohammed Y. Areeshi, Raju K. Mandal, Sajad A. Dar, Abdulrahman M. Alshahrani, Aqeel Ahmad, Arshad Jawed, Mohd Wahid, Mohtashim Lohani, Aditya K. Panda, Shafiul Haque

**Affiliations:** 1 Research and Scientific Studies Unit, College of Nursing and Allied Health Sciences, Jazan University, Jazan 45142, Saudi Arabia; 2 Department of Microbiology, University College of Medical Sciences and GTB Hospital (University of Delhi), Delhi 110095, India; 3 Department of Biochemistry, College of Medicine, Shaqra University, Shaqra 11961, Saudi Arabia; 4 Life Sciences Division, GE Healthcare Life Sciences, Gurgaon 122009, India; 5 Centre for Life Sciences, Central University of Jharkhand, Ranchi 835205, Jharkhand, India

**Keywords:** genetic model, Meta-analysis, polymorphism, pulmonary tuberculosis, VDR

## Abstract

*Bsm*I (rs1544410) polymorphism located in intron 8 at the 3′-end of the vitamin D receptor (VDR) gene is known to be involved in the regulation of mRNA stability. Many studies evaluated the possible correlation between VDR *Bsm*I polymorphism and the risk of pulmonary tuberculosis (PTB), and reported conflicting results. In the present study, an updated meta-analysis was performed to evaluate the above-said association. PubMed, Embase, and Google Scholar web-databases were searched for the relevant studies and a meta-analysis was performed by calculating pooled odds ratios (ORs) and 95% confidence intervals (95% CIs) for all the genetic models. A total of 19 studies comprising 3644 controls and 2635 cases were included in the present study. Overall no association of PTB in allelic contrast (b compared with B: *P*=0.285; OR =0.909, 95% CI =0.762–1.083), homozygous (bb compared with BB: *P*=0.881; OR =0.975, 95% CI =0.700–1.359), heterozygous (bB compared with BB: *P*=0.834; OR =1.017, 95% CI =0.872–1.185), dominant (bb compared with BB + Bb: *P*=0.451; OR =0.954, 95% CI =0.843–1.079) and recessive (bb + Bb compared with BB: *P*=0.983; OR =1.002, 95% CI =0.868–1.156) genetic models in comparison with wild-type allele and genotype BB were observed. However, variant allele (b compared with B: *P*=0.001; OR =2.289, 95% CI =1.661–3.154) showed increased risk of PTB in Asians. In conclusion, VDR *Bsm*I polymorphism is not a risk factor for PTB in overall population. However, this polymorphism may be interrelated to an increased risk of PTB amongst Asians.

## Introduction

Pulmonary tuberculosis (PTB) is the most common type of active TB that severely affects the lungs. PTB is still a leading cause of deaths across the world [1]. Regardless of the successful initiation and expansion of DOTS (directly observed treatment, short-course) and the Stop TB Strategy in most parts of the world, early and accurate identification of TB infection remains a serious challenge. In general, the risk of developing TB in infected individuals ranges from 5 to 10% [2], which indicates that besides the *Mycobacterium tuberculosis* strain itself, the host genetic factors may also determine the differences in the host’s susceptibility toward TB [3]. Unfortunately, the etiology of TB is very vague and genetic variations play a crucial role in regulating the genes of immune response that impart the susceptibility to developing active TB [4]. Previously, it had been reported that the contribution of genetic factors to the phenotypic variations and immune responses in the population infected with TB ranged up to 71% [5]. In the recent years, the immune-related genes have drawn significant interest of researchers majorly due to the hypothesized association between the immunity and TB.

Vitamin D_3_ acts as a modulator that activates the monocytes and stimulates the body’s immune system [6]. The active form of vitamin D plays a key role in monocyte/macrophage activation via vitamin D receptor (VDR) and it has been shown that vitamin D is one of the few mediators reported to impair the growth of *M. tuberculosis* in the macrophages [7]. VDR gene is positioned on chromosome 12q and has numerous common allelic variants. It comprises 15 exons (protein-coding exons: 2–9 and untranslated exons: 1a–1f) and two alternate promoter regions [8]. VDR gene contains multiple allelic variants and some of them may lead to alterations in the VDR function and are considered a risk factor for immune-mediated TB development. The majority of polymorphisms in VDR gene are found to be in the regulatory regions such as the 5′-promoter area and the 3′-UTR region rather than the coding exons [9]. Variation in the regulatory region of the gene may alter protein sequence and could result in drastic functional effects, such as changes in the affinity for the ligand or binding to the DNA. One of the most frequently studied polymorphisms is *Bsm*I, which is present on intron 8 bound to the 3′-UTR of VDR gene, is related to the regulation of mRNA stability and half-life [10]. This polymorphism in the 3′-UTR region of the gene appears to be in strong linkage disequilibrium (LD), and the haplotype frequencies are associated with increased level of VDR [11]. *Bsm*I polymorphism has been shown to be related with VDR activity or expression [12,13]. Studies have reported that allelic differences in VDR gene including *Bsm*I polymorphism may affect gene expression through regulation of mRNA stability [14,15]. Altered *VDR* mRNA expression has been correlated with variant genotypes of the VDR gene [9].

**Figure 1:**
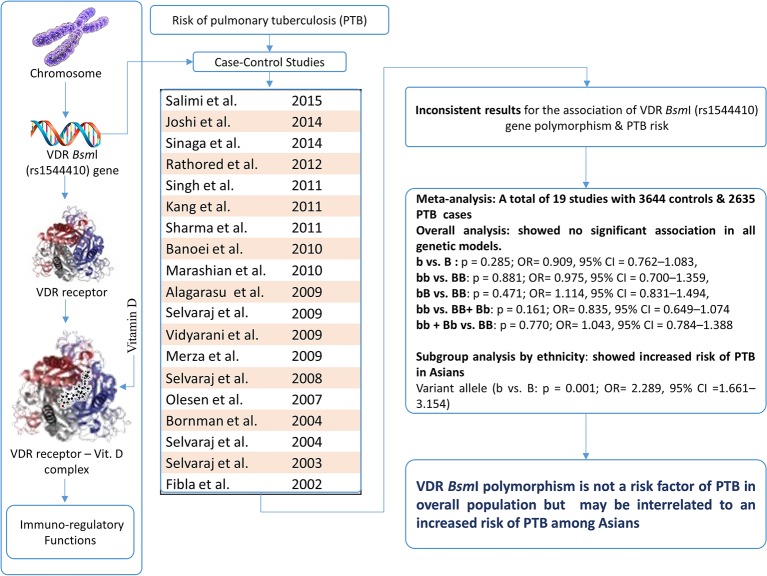
The meta-analysis performed to evaluate the association of VDR *Bsm*I (rs1544410) gene polymorphism and the risk of PTB.

Keeping the biological significance of this genetic variant in view, several subsequent reports have been published mentioning the impact of VDR *Bsm*I (rs1544410) polymorphism on the susceptibility of PTB in different populations [16–34]. The results of most of the published reports are inconclusive and inconsistent, and still there is no clear answer about whether the specific VDR *Bsm*I genotype is associated with PTB and contributes to higher/reduced risk of PTB. In addition, the individual studies might have been underpowered to detect the overall effects. Most of the published studies are facing the problems of limited sample size, ethnic diversity, and subsequently suffer too low power to detect the actual effects that may actually exist. In view of sufficient amount of accumulated data, we deemed it is logical to perform a quantitative synthesis of the evidence by applying meta-analysis and provide more precise conclusion of the previous findings. Hence, in the present study, a meta-analysis based on a total of 19 independent studies was performed to evaluate the precise association of VDR *Bsm*I gene polymorphism and risk of developing PTB. A meta-analysis is a powerful statistical tool for studying cumulative data from the independent studies, where individual sample sizes are small and bear low statistical power [35]. The schematic representation of the entire study has been given as Figure 1 (graphical abstract).

## Materials and methods

### Identification and eligibility of the relevant studies

A carefully planned literature search was carried out using PubMed (Medline), Embase, and Google Scholar web databases encompassing all the research articles published with a combination of the following keywords: ‘VDR’, ‘Vitamin D receptor’ gene (‘polymorphism’ OR ‘mutation’ OR ‘variant’) AND ‘tuberculosis’ OR ‘TB’ OR ‘pulmonary tuberculosis’ OR ‘PTB’ AND ‘susceptibility’ OR ‘risk’ (last updated on June 2016). All studies that showed potential relevance for genetic association were weighed by examining their titles and abstracts. All the published studies matching with the above-said eligibility criteria were obtained and tested for their eligibility for incorporation in the present meta-analysis.

### Inclusion and exclusion criteria

In order to minimize heterogeneity and facilitate the precise elucidation of the results, studies included in the present meta-analysis study had to pass the following criteria: (i) all included studies must have appraised the association between VDR *Bsm*I gene polymorphism and PTB risk, (ii) adopted a case–control study design, (iii) clearly described confirmed PTB patients and TB-free healthy controls, (iv) have available genotype frequency in the ‘cases’ and in the ‘controls’, (v) published in the English language, and (vi) data collection and analysis methodology should be valid from the statistical point of view. Also, when the case–control was reported in more than one research article using the same case series, we selected the only research study that included the largest number of the subjects. The major reasons for the exclusion of the studies from this meta-analysis were: (i) overlapping of the data, (ii) case-only studies, and (iii) review articles.

### Data extraction

For each retrieved study, the procedural quality assessment and data extraction were independently summarized in duplicate copies by the two independent investigators (Sajad A. Dar & Raju K. Mandal) following a standard protocol. During the data extraction process, data-collection form were used to ensure the accuracy of the collected data by stringently following the pre-set inclusion/exclusion criteria as mentioned above, and sequential exclusion of the unsuitable studies. In case of disagreement between the above mentioned two investigators on any item related with the data collected from the selected studies, the issue was fully discussed and deliberated with the investigators to reach a final consensus. Also, in case of failure in achieving consensus between the two investigators, the consensus was attained by following an open argument with the adjudicator (S.H.). The main characteristics abstracted from the retrieved publications comprised the name of the first author, the country of origin, publication year, the number of cases and controls, source of cases and controls, study type, genotype frequencies, and association with PTB.

### Quality assessment score of the selected studies

The methodological quality evaluation of the selected studies was carried out independently by the two investigators (SAD & RKM) by using the Newcastle–Ottawa Scale (NOS) of quality score assessment [36]. The NOS quality assessment criteria included three major aspects; (i) subjects’ selection: 0–4 points, (ii) comparability of the subjects: 0–2 points, and (iii) clinical outcomes: 0–3 points. Selected case–control studies that gained five or more stars were rated moderate to good quality [37].

### Statistical analysis

Pooled odds ratios (ORs) and their respective 95% confidence intervals (95% CIs) were calculated to assess the relation between the VDR *Bsm*I gene polymorphism and PTB risk. Heterogeneity belief was scrutinized by the chi-square based Q-test [38]. The significance level (*P*-value <0.05) for the Q-test revealed a lack of heterogeneity amongst the selected studies. Fixed/random effects model was used to pool ORs [39,40]. Additionally, *I^2^* statistics were employed to calculate interstudy variability ranging between 0 and 100%, wherein, 0% indicated no observed heterogeneity and larger values indicate an increasing degree of heterogeneity [41]. The Hardy–Weinberg equilibrium (HWE) in the control group was evaluated using chi-square test. Funnel plot asymmetry was calculated by the Egger’s linear regression test, which is a linear regression methodology to calculate the funnel plot asymmetry on the natural logarithm scale of the OR. The significance of the intercept was determined by the *t*test (statistically significant publication bias was indicated by *P*-value <0.05) [42]. The complete statistical analysis for this meta-analysis was performed by using the comprehensive meta-analysis (CMA) version 2 software program (BioStat Inc., U.S.A.). The CMA V2 software program has several positive aspects as compared with other programs available for computing the pooled analyses. A comparison of meta-analysis programs was obtained by using url link http://meta-analysis.com/pages/comparisons.html.

## Results

### Characteristics of the published studies

**Figure 2:**
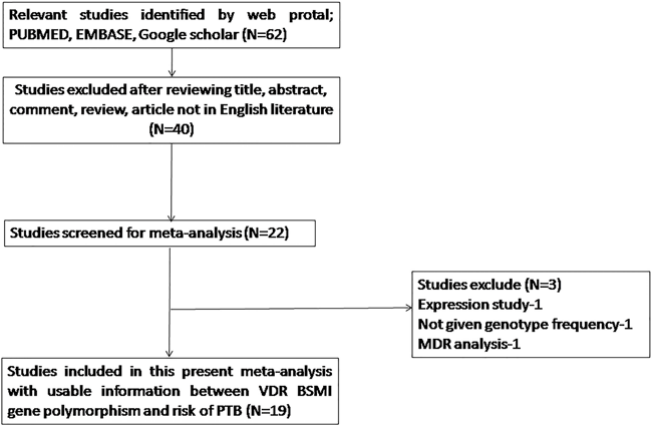
PRISMA flow diagram showing he selection process (inclusion/exclusion) of the studies dealing with VDR *Bsm*I (rs1544410) gene polymorphism and PTB risk

A total of 62 hits were obtained during the literature search performed on PubMed (Medline), Google Scholar, and Embase online web-databases. All the retrieved hits/articles were scrutinized by reading their titles, abstracts, and the full texts for the possibly relevant publications. Additionally, the articles were further checked for their suitability for the present meta-analysis (Figure 2: PRISMA 2009 Flow Diagram). Likewise, the references listed in the retrieved articles were also reviewed for other possible pertinent articles. Published studies of VDR polymorphism to predict survival and considering it as an indicator for response to therapy were excluded from this meta-analysis. Retrieved studies that reported the levels of *VDR* mRNA or protein expression and review articles were excluded from this pooled analysis. A very stringent criterion was followed in articles’ search, only case–control or cohort design studies with the frequencies of all the three genotypes were included. After thorough analysis and following the stern inclusion and exclusion criteria, 19 originally published studies were found eligible and included in the present pooled study (Table 1). A comprehensive flowchart of the selection process of the studies is shown in Figure 2 as prescribed by PRISMA Flow Diagram. The distribution of genotypes, HWE, *P*-values in the controls, and the susceptibility to PTB have been shown in Table 2. All the selected studies (19 in number) were examined for the overall quality following the NOS and most of the studies (>80%) scored five stars or more, and indicated modest to decent quality (Table 3).

**Table 1:** Main characteristics of all the 19 studies summarized for the present meta-analysis

First authors and Refs.	Year	Country of origin	Study design	Ethnicity	Cases	Controls	Selection of controls	Source of genotyping
Salimi et al. [16]	2015	Iran	HB	Asian	120	131	No clinical symptoms and history of PTB, chest X-ray, C-reactive protein (CRP) was measured	PCR-RFLP
Joshi et al. [17]	2014	India	HB	Asian	110	115		PCR-RFLP
Sinaga et al. [18]	2014	Indonesia	HB	Asian	76	76	Chest X-ray, no history of TB, tuberculin test	PCR-RFLP
Rathored et al. [19]	2012	India	HB	Asian	338	205	No clinical history of TB, chest X-ray, peripheral blood count, liver and kidney function	PCR-RFLP
Singh et al. [20]	2011	India	HB	Asian	101	225	No clinical symptoms and history of TB, normal CXR findings and PPD skin test results <5 mm	PCR-RFLP
Kang et al. [21]	2011	Korea	HB	Asian	150	83	No clinical history of TB	PCR-RFLP
Sharma et al. [22]	2011	India	HB, PB	Asian	261	1053	No clinical history of TB	PCR-RFLP
Banoei et al. [23]	2010	Iran	HB	Asian	60	62	No clinical history of TB	PCR-RFLP
Marashian et al. [24]	2010	Iran	HB	Asian	164	50	Chest X-ray, three serial sputum smear tests	PCR-RFLP
Alagarasu et al. [25]	2009	India	HB	Asian	105	146	No clinical history of TB	PCR-RFLP
Selvaraj et al. [26]	2009	India	HB	Asian	65	60	No clinical history of TB	PCR-RFLP
Vidyarani et al. [27]	2009	India	HB	Asian	40	49	No clinical history of TB	PCR-RFLP
Merza et al. [28]	2009	Iran	HB	Asian	117	60	No clinical history of TB, PPD skin test	PCR-RFLP
Selvaraj et al. [29]	2008	India	HB	Asian	51	60	No clinical history of TB	PCR-RFLP
Olesen et al. [30]	2007	West Africa	HB	African	320	345	No clinical history of TB	TaqMan
Bornman et al. [31]	2004	West Africa	PB	African	343	634	No clinical history of TB	PCR-RFLP
Selvaraj et al. [32]	2004	India	HB	Asian	46	64	No clinical history of TB	PCR-RFLP
Selvaraj et al. [33]	2003	India	HB	Asian	120	80	No clinical history of TB	PCR-RFLP
Fibla et al. [34]	2002	Spain	PB	European	48	146	No clinical history of TB, negative test of TB	Not mentioned

Abbreviations: HB, hospital based; PB, population based.

**Table 2:** Distribution of VDR *Bsm*I gene polymorphism of studies included in the present meta-analysis

Authors, year of publication, and Refs.	Control				Case				HWE
	Genotype			Minor allele	Genotype			Minor allele	
	BB	Bb	bb	MAF	BB	Bb	bb	MAF	p-value
Salimi et al. (2015) [16]	39	70	22	0.43	31	66	23	0.46	0.31
Joshi et al. (2014) [17]	55	37	23	0.36	35	58	17	0.41	0.00
Sinaga et al. (2014) [18]	2	18	56	0.85	0	52	24	0.65	0.70
Rathored et al. (2012) [19]	51	108	46	0.48	72	204	142	0.58	0.43
Singh et al. (2011) [20]	57	134	34	0.44	32	52	17	0.42	0.00
Kang et al. (2011) [21]	0	8	75	0.95	2	13	135	0.94	0.64
Sharma et al. (2011) [22]	274	734	211	0.47	60	89	37	0.43	0.00
Banoei et al. (2010) [23]	31	26	5	0.29	13	27	20	0.55	0.88
Marashian et al. (2010) [24]	0	29	21	0.71	23	86	55	0.59	0.00
Alagarasu et al. (2009) [25]	39	62	45	0.52	37	39	27	0.45	0.07
Selvaraj et al. (2009) [26]	16	23	21	0.54	27	22	16	0.41	0.07
Vidyarani et al. (2009) [27]	15	13	21	0.56	16	14	10	0.42	0.00
Merza et al. (2009) [28]	13	21	26	0.60	7	67	43	0.65	0.03
Selvaraj et al. (2008) [29]	16	17	27	0.59	23	16	12	0.39	0.00
Olesen et al. (2007) [30]	38	152	152	0.66	33	141	146	0.67	1.00
Bornman et al. (2004) [31]	39	208	387	0.77	20	108	215	0.78	0.12
Selvaraj et al. (2004) [32]	18	27	19	0.50	16	24	6	0.39	0.21
Selvaraj et al. (2003) [33]	32	29	19	0.41	42	56	22	0.41	0.02
Fibla et al. (2002) [34]	15	80	41	0.59	3	32	13	0.60	0.00

Abbreviation: MAF, minor allele frequency.

**Table 3:** Quality assessment conducted according to the NOS for all the studies included in the meta-analysis

First author and year	Quality indicators		
	Selection	Comparability	Exposure
Salimi et al. (2015) [16]	***	**	**
Joshi et al. (2014) [17]	***	*	**
Sinaga et al. (2014) [18]	***	**	**
Rathored et al. (2012) [19]	***	**	**
Singh et al. (2011) [20]	***	*	**
Kang et al. (2011) [21]	**	*	***
Sharma et al. (2011) [22]	***	*	**
Banoei et al. (2010) [23]	**	*	**
Marashian et al. (2010) [24]	**	*	***
Alagarasu et al. (2009) [25]	***	*	**
Selvaraj et al. (2009) [26]	**	*	**
Vidyarani et al. (2009) [27]	**	*	**
Merza et al. (2009) [28]	**	*	**
Selvaraj et al. (2008) [29]	***	*	**
Olesen et al. (2007) [30]	***	*	**
Bornman et al. (2004) [31]	***	*	**
Selvaraj et al. (2004) [32]	***	*	**
Selvaraj et al. (2003) [33]	***	*	**
Fibla et al. (2002) [34]	***	*	***

The number of asterisks indicates the number of criteria each publication is fulfilling for quality of ‘Selection’, ‘Comparability’ and ‘Exposure’. A study with a collective score of five asterisks or more is rated as good quality study.

### Publication bias

The Begg’s funnel plot and Egger’s test were carried out to estimate the publication bias amongst the studies included in this meta-analysis (Table 4). The emergence of the shape of the funnel plots and the results of Egger’s test have not shown the evidence of publication bias for all the comparison models (b compared with B, bb compared with BB, bB compared with BB, bb + bB compared with BB, and bb compared with BB + Bb) (Table 4).

**Table 4:** Overall statistics to test publication bias and heterogeneity of this meta-analysis

Comparisons	Egger’s regression analysis			Heterogeneity analysis			Model used for this meta-analysis
	Intercept	95% CI	*P*-value	Q-value	*P*_heterogeneity_	*I^2^* (%)	
b compared with B	–1.97	–4.57 to 0.62	0.12	73.158	0.000	75.396	Random
bb compared with BB	–0.640	–2.63 to 1.35	0.50	52.043	0.000	65.413	Random
Bb compared with BB	0.818	–1.08 to 2.72	0.37	52.945	0.000	66.002	Random
bb + Bb compared with BB	0.277	–1.77 to 2.33	0.77	57.857	0.000	68.880	Random
bb compared with BB + Bb	–1.87	–4.00 to 0.26	0.081	63.511	0.000	71.658	Random

### Test of heterogeneity

The Q-test and *I^2^* statistics were applied to check heterogeneity amongst the studies included in this meta-analysis. When the Q-test of heterogeneity was found significant in all the genetic models, i.e. allele (b compared with B), homozygous (bb compared with BB), heterozygous (bB compared with BB), recessive (bb + Bb compared with BB), and dominant (bb compared with BB + Bb), we applied random-effects model to analyze the combined OR and 95% CI for all the comparisons (Table 4).

### Meta-analysis of VDR *Bsm*I polymorphism and PTB susceptibility

**Figure 3:**
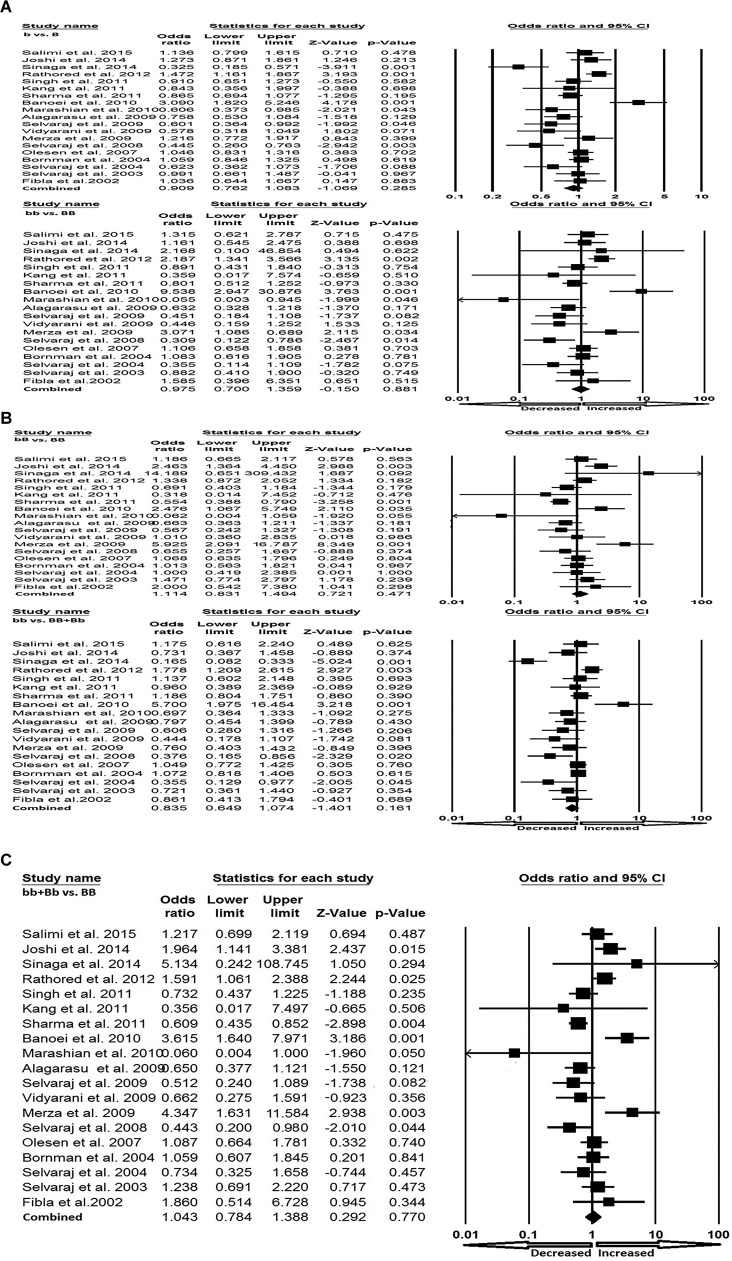
Forest plot. (**A**) Forest plot for overall analysis (allele: b compared with B; homozygous: bb compared with BB) showing OR with 95% CI to evaluate the association of the VDR *Bsm*I (rs1544410) gene polymorphism and PTB risk. (**B**) Forest plot for overall analysis (heterozygous: bB compared with BB; dominant: bb compared with BB + Bb) showing OR with 95% CI to evaluate the association of the VDR *Bsm*I (rs1544410) gene polymorphism and PTB risk. (**C**) Forest plot for overall analysis (recessive: bb + Bb compared with BB) showing OR with 95% CI to evaluate the association of the VDR *Bsm*I (rs1544410) gene polymorphism and PTB risk.

All the selected 19 studies were pooled together and resulted into 3644 controls and 2635 PTB cases. Based on heterogeneity, random-effects model was employed to assess the overall association between the VDR *Bsm*I gene polymorphism and the risk of PTB. Variant allele (b compared with B: *P*=0.285; OR =0.909, 95% CI =0.762–1.083), homozygous (bb compared with BB: *P*=0.881; OR =0.975, 95% CI =0.700–1.359), heterozygous (bB compared with BB: *P*=0.471; OR =1.114, 95% CI =0.831–1.494), dominant (bb compared with BB + Bb: *P*=0.161; OR =0.835, 95% CI =0.649–1.074) and recessive (bb + Bb compared with BB: *P*=0.770; OR =1.043, 95% CI =0.784–1.388) genetic models did not show any risk of the occurrence of PTB in response to the VDR *Bsm*I gene polymorphism as compared with the wild-type homozygous BB genotype (Figure 3A–C).

### Sensitivity analysis

**Figure 4:**
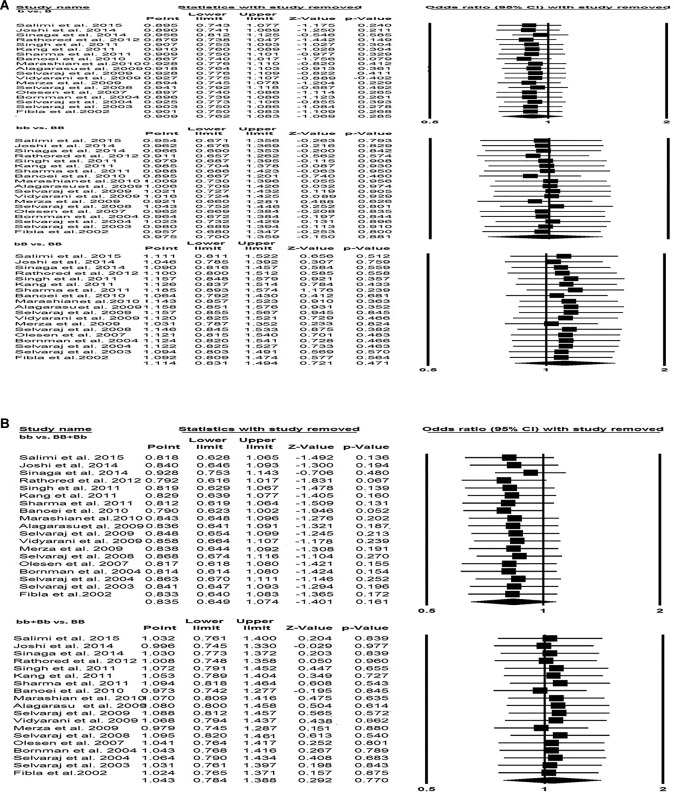
Sensitivity analysis. (**A**) Sensitivity analysis (allele: b compared with B; homozygous: bb compared with BB; heterozygous: bB compared with BB). (**B**) Sensitivity analysis (dominant: bb compared with BB + Bb; recessive: bb + Bb compared with BB).

Sensitivity analysis was performed to examine the effect of each individual study on the pooled OR by serially deleting one single study each time in succession. The results demonstrated that no individual study influenced the pooled OR substantially, hence results of the present meta-analysis were relatively stable and credible (Figure 4A,B).

### Subgroup analysis

Subgroup analysis based on the ethnicity was performed to detect any relationship between VDR *Bsm*I gene polymorphism and PTB risk in Asian population. This subgroup pooled analysis included 17 studies from Asian populations.

### Asian population

**Figure 5:**
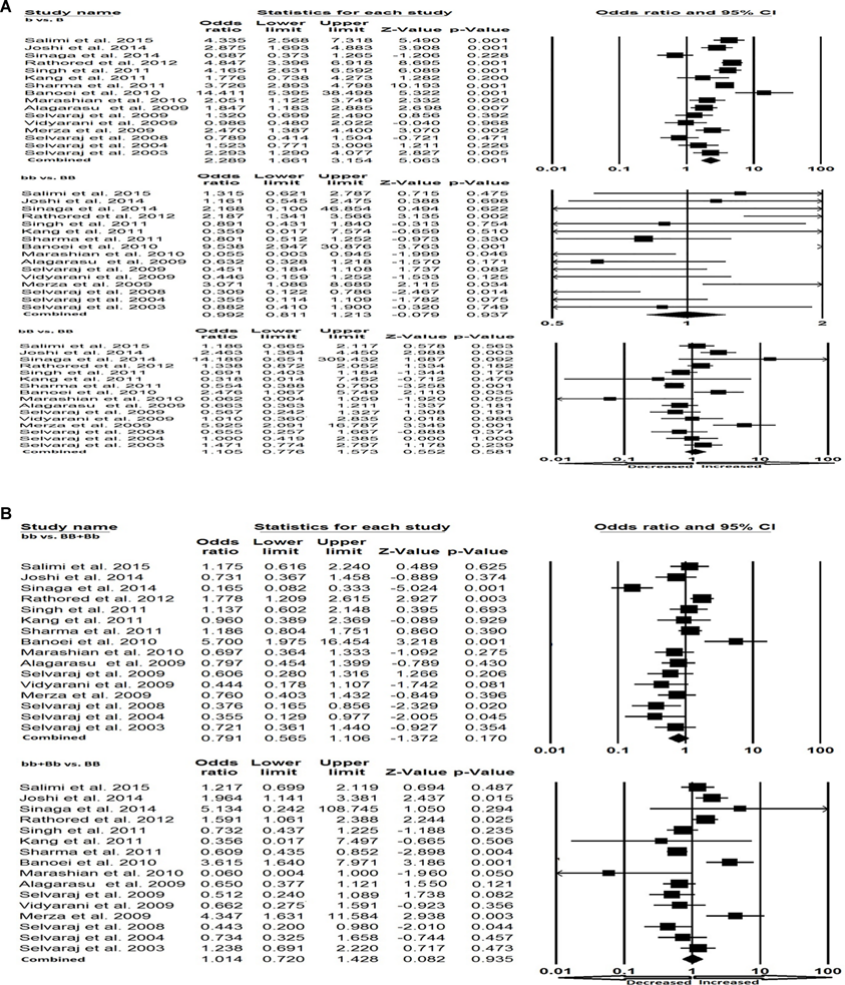
Forest plot. (**A**) Forest plot for the Asian population (b compared with B; bb compared with BB; bB compared with BB) showing OR with 95% CI to evaluate the association of the VDR *Bsm*I (rs1544410) gene polymorphism and PTB risk. (**B**) Forest plot for the Asian population (bb compared with BB + Bb; bb + Bb compared with BB) showing OR with 95% CI to evaluate the association of the VDR *Bsm*I (rs1544410) gene polymorphism and PTB risk.

In Asian population, the Q-test of heterogeneity was significant and we performed the analysis using random-effect models in all the genetic models (Table 5). We found statistically significant increased risk of PTB in Asian population in allele (b compared with B: *P*=0.001; OR =2.289, 95% CI =1.661–3.154) genetic model (Figure 5A). Whereas, in other genetic models, i.e. homozygous (bb compared with BB: *P*=0.937; OR =0.992, 95% CI =0.811–1.213), heterozygous (bB compared with BB: *P*=0.581; OR =1.105, 95% CI =0.776–1.573), dominant (bb compared with BB + Bb: *P*=0.170; OR =0.791, 95% CI =0.565–1.106) and recessive (bb + Bb compared with BB: *P*=0.935; OR =1.014, 95% CI =0.720–1.428) did not show any risk of PTB associated with VDR *Bsm*I polymorphism (Figure 5A,B).

**Table 5:** Statistics to test publication bias and heterogeneity in Asian population

Comparisons	Egger’s regression analysis			Heterogeneity analysis			Model used for this meta-analysis
	Intercept	95% CI	*P*-value	Q-value	*P*_heterogeneity_	*I^2^* (%)	
b compared with B	–3.20	–6.66 to 0.24	0.06	87.68	0.000	82.89	Random
bb compared with BB	–0.778	–3.15 to 1.59	0.493	51.443	0.000	70.842	Random
Bb compared with BB	0.740	–1.482 to 2.962	0.486	51.842	0.000	71.066	Random
bb + Bb compared with BB	0.154	–2.246 to 2.556	0.891	56.748	0.000	73.56	Random
bb compared with BB + Bb	–2.887	–6.338 to 0.562	0.094	61.787	0.000	75.723	Random

## Discussion

Lately, genetic susceptibility to PTB has led to increasing awareness of the study of polymorphisms of genes involved in PTB infection. It resulted in the investigation of a number of candidate genes to analyze the possible connection between modulations of PTB risk across various populations. Vitamin D has an immunomodulatory action that activates monocytes and suppresses lymphocyte proliferation, immunoglobulin production, cytokine synthesis, and plays a key role in human innate immunity to certain infectious agents including *M. tuberculosis* [10]. Vitamin D restricts the growth of *M. tuberculosis* in macrophages through the production of the antimicrobial peptide, cathelicidin [43], which is important for the body’s defense system against the development of PTB. The VDR gene encodes a ligand-activated transcription factor mediating multiple actions of vitamin D, including calcium homeostasis, cell differentiation, cell growth, modulation of the immune response, and activation of monocyte macrophages [44]. The molecular and functional consequences of the VDR polymorphisms are important to fully appreciate their significance and to recognize their potential clinical implications. Therefore, VDR *Bsm*I polymorphism is considered to be one of the possible factors, which may contribute to PTB predisposition.

Till date, various reports have been published that evaluated the possible association of VDR *Bsm*I gene polymorphism and PTB development, but the findings from different known studies were inconsistent and contradictory. Hence, pooled analysis with sufficient power was needed to summarize the independent individual studies. In the present meta-analysis, we aimed to obtain summary estimates for the strength of the VDR *Bsm*I gene polymorphism and PTB risk from 19 case–control studies, as pooling of the data from individual studies has the advantage of reducing the random errors [45]. Also, most of the included studies scored five or more stars in NOS quality assessment score criteria and suggested good to moderate quality by clearly stating about the sample size, genotype, inclusion criteria of leprosy patients, and healthy controls.

In the present study, associations for the allele contrast, dominant and recessive models were evaluated, and found no evidence of PTB development risk against VDR *Bsm*I gene polymorphism in 3644 controls and 2635 PTB cases. It is possible that the analyzed variant (i.e. VDR *Bsm*I) does not act as a primary susceptibility polymorphism and may be interacting with other causative germ-line polymorphisms found in LD and inhibits VDR functions. However, the present meta-analysis clearly demonstrates that the contribution of VDR *Bsm*I polymorphism to PTB is perhaps very low. Previously, Rashedi et al. [46] also reported no relationship between plasma vitamin D levels and frequency of *Bsm*I gene polymorphism in TB patients. Previous report of Wu et al. [47] reported that VDR *Bsm*I gene polymorphism is associated with a significant decreased TB risk, especially in Asian population, which is inconsistent with several previous independent studies. Also, they found some contradictory results of increased TB risk due to bb genotype in Iranian population and suggested that this may derive from different experimental designs or methods, and warranted for further investigation. In comparison with previously published reports, the present study has major improvements, as it has included only specific PTB cases of relevant published studies. When we studied Asian population separately, we found strong evidence that variant allele b confers susceptibility to PTB in Asians. This finding may help to explain the individual differences in the susceptibility to PTB. A study by Gao et al. [48] reported that *Bsm*I polymorphism is associated with TB risk only in Asian population. However, more experimental studies with larger sample size or alternative methods must be applied for further investigation to verify such findings as only the mutant allele showed significant outcome.

As it is established that TB is a complex, multifactorial disease influenced by both environmental and genetic factors [5], hence, a single genetic variant is normally insufficient to prophesize the susceptibility toward this dreadful disease. The important feature of this gene polymorphism is that their occurrence can vary sufficiently amongst different races or ethnic populations.

Despite the outcomes attained from this meta-analysis and prior to reaching a final conclusion, limitations of this meta-analysis should also be acknowledged. First, we found significant heterogeneity in the overall analysis. Many factors might contribute to this heterogeneity, variation in patients’ characteristics might be an important source of heterogeneity. Some studies used matched controls (e.g. age and sex matched), while, other studies did not perform matching. Second, the reports published only in the English language were considered in the present study. Third and the most important limitation is that the studies indexed by the selected electronic web-databases (i.e., PubMed, Embase, and Google Scholar) were searched for this pooled data analysis. The possibilities are there that some pertinent articles might have been published in other languages and/or indexed in other databases (which are not known to us), may have been missed. The fourth limitation, since the relevant complete data are not available most of the time, hence, we failed to adjust the confounding factors, such as, age, sex, HIV status, and TB severity in this meta-analysis. The fifth constraint was that we were unsuccessful in computing the gene and environmental interactions because of lack of sufficient information in the primary studies.

Despite the above-mentioned drawbacks, there are some strengths of our meta-analysis that support the robustness of the present results. First, this meta-analysis involved a large set of harmonized individual level data from 19 independent studies, which can provide enough statistical power to confirm our results. Second, since funnel plot and Egger’s test indicated no publication bias, thus all the results are statistically robust as per the sensitivity analysis. Also, all the included studies were of good to modest quality fulfilling the preset needful criteria as tested by NOS quality assessment scale. Although, plenty of meta–analyses have been performed in the past considering various case–control studies analyzing this relationship selected from various databases and concluded accordingly [47–49]. For example, a recent pooled study by Lee and Song [49], based on eight studies reported that *Bsm*I polymorphism is not associated with risk of PTB. Due to limited database selection, each pooled analysis misses some of the relevant studies. The research study available in one database is not necessarily present in the other one. Hence, each meta–analysis faces limitation of database selection. Likewise, in the present meta–analysis, most reliable databases were considered and some of the missing studies which were not included in other published studies [46] were incorporated and concluded accordingly.

## Conclusion

In conclusion, this meta-analysis objectively evaluated various independently published studies dealing with the relationship of VDR *Bsm*I gene polymorphism with PTB risk and found no evidence of association between VDR *Bsm*I polymorphism and overall PTB risk. Interestingly, we found that variant allele of *Bsm*I polymorphism may contribute to PTB pathogenesis and may help in explaining the individual differences in the susceptibility of PTB in the Asian population. These results suggest that VDR *Bsm*I gene polymorphism is unlikely to be an important risk factor for specific infectious PTB disease. Furthermore, large, well-designed epidemiological studies are needed to understand the roles of VDR *Bsm*I gene polymorphism in the pathogenesis of PTB, especially in the Asian population.

## Abbreviations

CI: confidence interval
CMA: comprehensive meta-analysis
HWE: Hardy–Weinberg equilibrium
LD: linkage disequilibrium
NOS: Newcastle–Ottawa scale
OR: odds ratio
PTB: pulmonary tuberculosis
VDR: vitamin D receptor
